# Effects on development and microbial community of shrimp *Litopenaeus vannamei* larvae with probiotics treatment

**DOI:** 10.1186/s13568-020-01041-3

**Published:** 2020-06-05

**Authors:** Ruixuan Wang, Zihan Guo, Yapeng Tang, Jiawei Kuang, Yafei Duan, Heizhao Lin, Shigui Jiang, Hu Shu, Jianhua Huang

**Affiliations:** 1 Shenzhen Base of South China Sea Fisheries Research Institute, Chinese Academy of Fishery Sciences, Shenzhen, 518121 China; 2 School of Food Engineering and Biotechnology, Hanshan Normal University, Chaozhou, 521041 China; 3 Key Laboratory of South China Sea Fishery Resources Exploitation & Utilization, Ministry of Agriculture and Rural Affairs; South China Sea Fisheries Research Institute, Chinese Academy of Fishery Sciences, Guangzhou, 510300 China; 4 Guangzhou University, Guangzhou, 510300 China; 5 California Baptist University, Riverside, CA 92504 USA

**Keywords:** *Litopenaeus vannamei*, Larvae, Probiotics, Development, Microbial community

## Abstract

Shrimp production is the second ranked of the most-traded production in these decades and the whiteleg shrimp *Litopenaeus vannamei* is the sixth most cultured species. Probiotics are alternative strategy for the promotion of growth and prevention of diseases in aquaculture. To confirm the effects of the probiotics on development and microbial community of *L. vannamei* larvae during different development stages, five kinds of probiotics (10^8^ ~ 10^9^ CFU/g) were added into the rearing environment of shrimp larvae, and the effects of probiotics on bacterial community and water quality, larval growth and immune index were determined from nauplius larval stage to post larval stage. Results suggested that probiotics treated groups showed larger survival rate than the control groups from Z1 stage to P5 stage. Lactobacillus could improve the larvae’s survival ability, especially in the larval stages M2, M3, P1, P5 stage. It was confirmed that probiotics could promote the growth and development of shrimp larvae and prevent the incomplete molting in their growing process, particularly for EM-treated group. Results suggested that all the probiotics-treated groups had shown significant decreasing trend in the quantity of vibrios, except for the SA-treated group. And different probiotics could inhibit vibrios during different life periods. Among these probiotics, LA, EM and PB had shown the best effects, including improving survival rate of the larvae, promoting the larval metamorphosis, reducing the quantity of vibrios and NH_4_-N and NO_2_-N levels, and increasing bacterial diversity.

## Key points

- The first study on bacterial flora in shrimp larvae at N6 ~ P5 stages with probiotics treated.
- Confirmed LA, EM and PB had the best effects on larvae’s survival, metamorphosis, and environmental quality.
- It was found that all the probiotics added externally did not become the dominant flora.

## Introduction

Aquaculture is an important industry, which was one of the important sources of food and nutrition, and of livelihoods for human (Chumpol et al. 2018). Shellfish aquaculture occupies an important position in the world economy. Shrimp production is the second ranked of the most-traded production in these decades in aquaculture. Marine shrimp production have increased from less than 10.000 metric tonnes in 1970 to more than 4.000.000 metric tonnes in 2014. Pacific white shrimp (*Litopenaeus vannamei*) has accounted for 80% of the production (FAO 2016). *L. vannamei*, *Penaeus monodon* and *Fenneropenaeus chinensis* are the three major shrimp species cultured in China. Quantities of farms for culturing *L. vannamei* have increased rapidly because of high economic value of *L. vannamei*. Probiotics, which contain potentially beneficial bacteria, are considered to be beneficial dietary supplements for human health several years ago (FAO 2001). Previous studies proved that probiotics play an important role in inhibiting pathogenic microorganisms, enhancing the host’s immunity and promoting their growth factors, including the enzymatic digestion (Verschuere et al. 2000; Krummenauer et al. 2014). Also, probiotics are used as an auxiliary method to reduce the use of antibiotics (Huerta-Rábago et al. 2019). Generally, probiotics contain three categories, including lactobacillus, bifidobacteria and some gram-positive cocci, such as the effective microorganisms (EM), bacillus (BA), lactobacillus (LA), photosynthetic bacteria (PB), saccharomyces (SA), etc. The beneficial effects of these probiotics include improving growth performance, enhancing the enzymatic contribution to nutrition, inhibiting of adherence and colonization of pathogenic bacteria in the digestive tract, and increasing haematological parameters and immune response (Ringø 2020).

During the breeding process of *L. vannamei*, quality of larvae is the key to the rates of survival, growth and metamorphosis, and will directly affect the success or failure during the breeding process. Recently, it has been reported that adding probiotics to the aquaculture water or the feed could improve the farming ecological environment and reduce aquatic diseases (Qiu et al. 2004; Adel et al. 2017; Nimrat et al. 2011; De la Banda et al. 2012). Moreover, it has suggested that probiotics could promote the larvae’s growth and development of fish or shrimp (as improving the utilization rate of nutrients), and improve the survival rate, also has made progress in purifying aquaculture ponds (Ringø 2020). It has been early proved that adding probiotics into the feed could accelerate the growth and development of Pacific oyster larvae (Douillet and Langdon 1994), and reduce the occurrence of diseases and improve the health status of aquatic animals (as they could improve the resistance of the body and inhibit the reproduction of pathogenic bacteria) (Nogam and Maeda 1992), reduce the pollution to the environment during the aquaculture process (Li et al. 2011). For example, appropriate PB could significantly reduce the ammonia nitrogen concentration in the cultured water for scallop (Wang et al. 1994), and significantly reduce nitrite nitrogen and chemical oxygen demand (COD) in the breeding water for Chinese mitten crab and enhance the animals’ metamorphosis rate (Yan et al. 2005). Otherwise, It was showed that probiotic could enhanced immune parameters, including the propo system, peroxinectin, penaeidin, thioredoxin, lectins, haemocyanin and crustin, and provided protection against white spot syndrome virus infection in Pacific white shrimp, such as *Bacillus*, which isolated from the gut of Chinese white shrimp (*F. chinensis*) (Chai et al. 2016). Therefore, probiotics have been defined as “live microorganisms, which, administered in adequate doses confer a health benefit to the host” (FAO 2001), while they are microorganisms that contribute to the balance of intestinal flora in animals.

Previous studies have reported the importance of bacterial communities in intestines for maintaining the metabolism and immunity of their host, as well as for evading the viral and bacterial diseases (Chaiyapechara et al. 2012; Zhang et al. 2014), which were always casused by the predominant opportunistic pathogens such as *Vibrio* species in the marine environments (Wang et al. 2015, 2016, 2018). And the high diversity of microorganisms plays an important role in stabilizing the aquatic system, included: maintenance of water quality (López-Elías et al. 2015), improving the nutrition, increasing culture feasibility and promotion the health of cultured organisms (Martínez-Córdova et al. 2017; Moreno-Arias et al. 2018; Aguilera-Rivera et al. 2014; Emerenciano et al. 2017). In the study of host-microbe interactions, the resident bacteria were considered prime contributors to long-lasting effects (Niu et al. 2012). Previously, many studies have been conducted to investigate the intestinal bacterial community in a wide range of vertebrates in aquaculture (such as zebrafish and rainbow trout) and invertebrates (such as black tiger shrimp and white shrimp) (Huang et al. 2016; Kim et al. 2007; Roeselers et al. 2011). Thus, it is essential to understand the bacterial community composition and alteration factors for the enhancement of aquaculture quality comprehensively (Oetama et al. 2016).

In commercial farms, it is difficult to develop controlled bioassays, and frequently, the results are not conclusive, although some companies use probiotics, but the effectiveness of probiotics are scarcely evaluated (Arias-Moscoso et al. 2018). In the present study, five kinds of probiotics (including EM, BA, LA, PB, SA) were added into the water environment for culturing the shrimp larvae, then the effects of probiotics on bacterial community and water quality, larval growth and the bacterial community were analyzed from nauplius (N) larval stage to post larval (P) stage, including N6, zoea (Z) larval stage (Z1, Z2, Z3), mysis (M) larval stage (M1, M2, M3), P1 and P5. Bacterial abundance and diversity in *L. vannamei* larvae and rearing water at different developmental stages were also analyzed. The present study will contribute to the generate information which can be useful for the commercial shrimp farms.

## Materials and methods

### Source of the probiotics

Five probiotics including the BA (the trade name is NanShuiLiSheng-01), EM (the trade name is Bioantai-01), LA (the trade name is Bioantai-02), PB (the trade name is NanShuiLiSheng-02) and SA (the trade name is NanShuiLiSheng-03) had been added to the plastic buckets respectively and then the plastic buckets were covering with black film for 8 h to activate the probiotics. The total number of BA over 10^9^ CFU/g, the total number of PB over 10^8^ CFU/mL, and the total number of SA over 10^9^ CFU/g, the above three probiotics belong to Guangzhou Xin Haley Biotechnology Co. Ltd., Guangzhou City, Guangdong, China. The total number of EM bacteria over 10^9^ CFU/g, and the total number of LA bacteria over 10^8^ CFU/g, the above two probiotics belong to Qingdao Bioantai Biotechnology Co. Ltd., Qingdao City, Shandong, China.

### Experimentation and samples collection

Shrimp *L. vannamei* larvae were collected from Shenzhen Base, South China Sea Fisheries Research Institute of Chinese Academy of Fishery Sciences (Shenzhen, China), larvae had been randomly assigned to a 500 L black plastic bucket containing 300 L seawater at a density of 300 nauplii/L. Before the larvae were put into the tested plastic buckets, five probiotics including the BA, EM, LA, PB and SA had been added to the plastic buckets, respectively and then the plastic buckets were covering with black film for 8 h to activate the probiotics. The concentration of probiotics was 0.005%. Control groups which only contained larvaes were analyzed synchronously. Every 5 parallels were carried out for experimental groups and the control groups. Seawater had been filtered through a sand filter, the water was not changed during the whole experiment period. The water temperature was maintained at 31.0–32.5 °C, salinity 28–30‰, pH 7.8–8.0 and continuous aeration of gas stone to maintain dissolved oxygen at 5.8–6.4 mg/L. Simultaneously, the rearing water samples were smeared on the thiosulfate citrate bile salts sucrose (TCBS) agar medium plates (three volume proper gradients, and three replicates for each volume). All the plates were then placed in incubators at 28 °C. After incubated with TCBS agar plates for 96 h, the number of bacterial colonies were counted and recorded (reportable numbers were limited to 30–300). Additionally, ammonia nitrogen (NH_4_-N) and nitrite (NO_2_-N) were also detected (according to the GB17378.4-2007).

Meanwhile, 1 L water sample collected from each bucket was filtered with 0.22 μm polycarbonate membrane filters (Millipore), so that all bacteria in the water samples were collected by the filter. And approximately 0.5 g larvae was collected from each bucket with a net, and the larvae were then washed three times with sterile seawater to remove microorganisms on their body surfaces. Then, all filters and larvae samples were rapidly frozen with liquid nitrogen for more than 10 min and then stored at − 80 °C for high-throughput sequencing and biochemical analysis. Three replicates for each group were analyzed.

### DNA isolation

The DNA of each sample was extracted by utilizing Mabio DNA Mini Kit (Guangzhou Jirui Gene Technology Co., LTD., Guangdong, China) according to manufacturer’s recommendations. Prior to the isolation protocol, each sample was preprocessed: the PBS-washed larvaes were centrifuged at 500 rpm for 4 min, the supernatant was separated and the sediments were rewashed twice. All of the collected supernatant was centrifuged at 13,000 rpm for 5 min. Then the supernatant was discarded and the sediments were rewashed twice and resuspended with 30 mL PBS. This procedure was repeated again and the final sediments were used for DNA isolation. The impurities which may hamper the PCR procedure were removed by utilizing AMPure. The integrities of gDNA were tested by agarose gel electrophoresis.

## 16S rRNA gene amplification

The intestinal samples were sent to Guangzhou JiRui Gene Technology Co. Ltd. (China) for extraction of DNA and PCR amplification by Illumina MiSeq Sequencing platform. PCR was performed from V3 ~ V4 variable regions of 16S rRNA to taxonomically identify the bacteria. The 16S rRNA gene with V3-V4 variable regions of PCR primers (F: CCTACGGRRBGCASCAGKVRVGAAT and R: GGACTACNVGGGTWTCTAATCC) with barcode on the forward primer were employed.

### Library preparation and sequencing

Sequencing libraries were generated using MetaVx™ Library prep Kit (South Plainfield, NJ, USA) following the manufacturer’s instructions (20–30 ng DNA was used to generate amplicons. V3 and V4 hypervariable regions of prokaryotic 16S rDNA were selected for generating amplicons and following taxonomy analysis. A panel of proprietary primers aimed at relatively conserved regions bordering the V3 and V4 hypervariable regions of bacteria and Archaea16S rDNA were designed.The V3 and V4 regions were amplified using forward primers containing the sequence “CCTACGGRRBGCASCAGKVRVGAAT” and reverse primers containing the sequence “GGACTACNVGGGTWTCTAATCC”. At the same time, indexed adapters were added to the ends of the 16S rDNA amplicons to generate indexed libraries ready for downstream NGS sequencing on Illumina Miseq. PCR reactions were performed in triplicate 25 μL mixture containing 2.5 μL of TransStart Buffer, 2 μL of dNTPs, 1 μL of each primer, and 20 ng of template DNA), and index codes were added. The library quality was assessed on the Qubit 2.0 Fluorometer (Invitrogen, Carlsbad, CA) and Agilent Bioanalyzer 2100 system. Then each of the libraries was performed with high-throughput sequencing on an Illumina MiSeq platform (PE300, Illumina, San Diego, CA, USA), and the paired-end reads were generated.

### Bioinformatics and statistical data analyses

The barcodes and primers were trimmed from the sequences and the short sequences < 200 bp were removed from the raw data. The sequences containing 6 bp and bigger homopolymer regions and ambiguous base calls were removed. Sequences were then denoised and chimeras were removed. Operational taxonomic units (OTUs) were identified after removal of sequences clustering at 3% divergence (97% similarity) with VSEARCH (1.9.6). OTUs were then taxonomically grouped and classified using BLASTn tool against a curated GreenGenes database and compiled into each taxonomic level into both “counts” and “percentage” files. Counts files contained the actual number of sequences while the percent files contained the relative (proportion) percentage of sequences within each sample that map the designated taxonomic classification. The representative sequence of OTUs were analyzed taxonomically, and the community composition of each sample was counted at different classification levels with Ribosomal Database Program (RDP) classifier. In order to compute alpha diversity, the OTUs tables were verified and random sampling of sequences were calculated, including the three metrics: Shannon estimated the species abundance; Observed species estimated the amount of unique OTUs found in each sample and Shannon index. Rarefaction curves were generated based on these three metrics. And the unweighted pair group method with arithmetic mean (UPGMA) evolutionary tree was constructed through hierarchical clustering.

## Results

### Survival rate of shrimp larvae during the development stage

At the beginning of the experiment, 90 thousand juveniles were put into each seedling barrel, and the survival rates in different stages were shown in Table 1. When the larvae reach the M stage, the phenomenon of sticky legs appeared. This was because there were too many nutrients in the rearing water. Sticky legs would cause the decline of the larvae’s vitality and their feeding capacity. The loss of feeding capacity would lead to malnutrition of the larvae and finally died. The present study showed that the survival rates of the experimental groups, including EM-, BA- and SA-treated groups, were higher than that of the control groups when the shrimp larvae developed from Z1 stage to P5 stage. With SPSS software, it suggested that the survival rate of LA-treated group was approximately 10% higher than that in the control groups (*P *< 0.05) in P5 stage, and PB-treated group (although survival rates were lower at Z1 stage) was 9% higher than that in the control groups in P5 stage (*P *< 0.05), respectively. It indicated that the five probiotic bacteria, especially the LA and PB, could obviously improve the larvae’s survival ability at the P stage, which was related to the seedling emergence rates.

**Table 1:** Survival rate of shrimp larvae in five treatment groups from N6 to P1 developmental phase (%, average value) (%)

Larval stages	Controls	Treatment groups				
		EM	BA	LA	PB	SA
Z1	91.33 ± 1.2	92.66 ± 1.2	92.33 ± 1.0	90.33 ± 1.6	88.33 ± 1.2	91.66 ± 0.4
Z2	82.66 ± 1.3	85.33 ± 0.4	85.66 ± 1.6	80.33 ± 1.2	82.33 ± 0.9	85.33 ± 0.9
Z3	70.66 ± 0.9	74.33 ± 1.1	77.00 ± 1.4	70.66 ± 1.6	71.66 ± 1.6	77.66 ± 1.2
M1	48.00 ± 1.6	53.00 ± 1.9	55.66 ± 1.0	49.50 ± 1.2	56.33 ± 1.3	59.00 ± 1.5
M2	37.50 ± 1.6	45.00 ± 1.5	46.00 ± 0.9	42.33 ± 1.6	45.33 ± 1.4	55.33 ± 1.6
M3	35.00 ± 0.7	41.33 ± 1.2	39.00 ± 0.6	40.33 ± 1.2	43.33 ± 1.5	51.33 ± 1.0
P1	31.50 ± 0.5	37.66 ± 1.2	33.00 ± 1.2	39.66 ± 0.4	41.00 ± 1.1	43.66 ± 1.6
P5	28.00 ± 0.3	35.66 ± 0.9*	30.00 ± 0.5	38.33 ± 2.0*	37.00 ± 1.4*	37.00 ± 1.0*

* *P* < 0.05

### Metamorphosis rate of shrimp larvae

At the beginning of the experiment, 90 thousand juveniles were put into each seedling barrel, and the metamorphosis rates in each stage were shown in Table 2. Generally, incomplete molting may appear in the shrimp larvae during the stage from Z3 to M1, which will make small part of the shell remain on the larvae, and result in incomplete metamorphosis. As shown in Table 2, compared with the control groups, the metamorphosis rate of each experimental group in different developmental stages were higher than that of the control group. It suggested that the metamorphosis rate of EM-treated or LA-treated groups were much higher than that in the control group respectively during every developmental stage, especially in P1 stage, the metamorphosis rate had increased by 10% or 9%, comparing with the control groups (*P *< *0.05*). It was proved that EM and LA could obviously facilitate the metamorphosis of the shrimp juveniles, and was conducive to the shrimp larvae’s cultivation. When reach P1 stage, other four probiotics could also enhance the metamorphosis of the larvae.

**Table 2:** Metamorphosis rate of shrimp larvae in five probiotics treatment groups from N6 to P1 developmental phase (%)

Larval stages	Controls	Treatment groups				
		EM	BA	LA	PB	SA
Z1	92.66 ± 2.0	93.66 ± 0.4	91.66 ± 0.9	92.00 ± 0.8	91.33 ± 2.0	93.33 ± 1.2
Z2	86.66 ± 1.6	88.66 ± 1.6	88.33 ± 1.6	83.66 ± 1.2	84.33 ± 1.2	88.33 ± 1.6
Z3	82.66 ± 1.2	89.33 ± 1.2	86.33 ± 1.2	79.66 ± 1.2	81.66 ± 2.0	86.00 ± 1.4
M1	77.50 ± 1.4	82.00 ± 0.9	81.00 ± 1.0	80.00 ± 0.7	76.33 ± 1.6	82.33 ± 2.0
M2	82.33 ± 1.4	85.33 ± 1.2	83.33 ± 0.4	84.00 ± 1.5	85.66 ± 1.6	88.00 ± 1.0
M3	80.00 ± 0.5	84.50 ± 0.7	86.50 ± 0.7	84.00 ± 1.4	79.33 ± 0.9	83.66 ± 0.7
P1	76.00 ± 1.2	86.00 ± 0.9*	83.33 ± 1.2	85.00 ± 0.9*	82.00 ± 1.4	83.00 ± 1.0

* *P* < 0.05

### Changes in the quantity of vibrios and the NH_4_-N, NO_2_-N

**Fig. 1:**
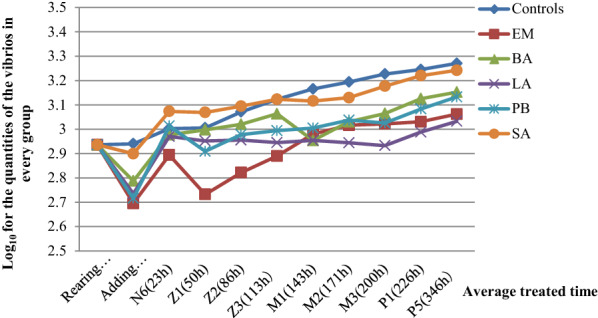
Changes in the quantities of vibrios in the water for culturing shrimp larvae from N6 to P1 developmental phase in each groups

It was showed that after adding probiotics, the quantity of vibrios in the water of the experimental groups had decreased, which was less than that in the control groups during N6 to Z1 (Fig. 1). There was a significant difference between the LA-treated groups and the control groups (*P *< 0.05), and between the EM-treated groups and the control groups (*P *< 0.05). Quantity of vibrios in the BA-treated and PB-treated groups were also much lower than control groups. It was showed that EM, LA, PB and BA had inhibitory effect on vibrios in the rearing water, while SA had no inhibitory effect.

It was showed that the NH_4_-N and NO_2_-N in each group showed a rising trend, but no significant increase was observed, which remained within the normal range (Table 3). Concentration of NH_4_-N and NO_2_-N in the rearing water with probiotics decreased to some extent. Concentrations of NH_4_-N and NO_2_-N in groups with EM, BA and PB were significantly lower than that in control group at the P5 stage (*P* < 0.05).

**Table 3:** Changes in ammonia nitrogen (NH_4_-N) and nitrite (NO_2_-N) in the rearing water from N6 to P1 developmental phase with five probiotics treatment (mg/L, n = 3)

	CK		EM		BA		LA		PB		SA	
	NH_4_-N	NO_2_-N	NH_4_-N	NO_2_-N	NH_4_-N	NO_2_-N	NH_4_-N	NO_2_-N	NH_4_-N	NO_2_-N	NH_4_-N	NO_2_-N
N6 (23 h)	0.046 ± 0.009	0.015 ± 0.003	0.045 ± 0.005	0.014 ± 0.002	0.040 ± 0.002	0.014 ± 0.001	0.046 ± 0.004	0.018 ± 0.002	0.045 ± 0.003	0.013 ± 0.001	0.051 ± 0.005	0.018 ± 0.003
Z1 (50 h)	0.060 ± 0.005	0.020 ± 0.002	0.058 ± 0.002	0.017 ± 0.002	0.054 ± 0.002	0.017 ± 0.001	0.061 ± 0.006	0.018 ± 0.002	0.064 ± 0.005	0.017 ± 0.001	0.058 ± 0.003	0.025 ± 0.003
Z2 (86 h)	0.070 ± 0.004	0.021 ± 0.003	0.066 ± 0.003	0.019 ± 0.003	0.065 ± 0.005	0.018 ± 0.002	0.074 ± 0.003	0.024 ± 0.003	0.070 ± 0.008	0.018 ± 0.001	0.062 ± 0.008	0.030 ± 0.001
Z3 (113 h)	0.079 ± 0.005	0.025 ± 0.002	0.073 ± 0.003	0.021 ± 0.002	0.07 ± 0.002	0.019 ± 0.001	0.082 ± 0.007	0.025 ± 0.003	0.075 ± 0.004	0.019 ± 0.002	0.077 ± 0.004	0.030 ± 0.003
M1 (143 h)	0.093 ± 0.009	0.030 ± 0.002	0.077 ± 0.003	0.024 ± 0.002	0.076 ± 0.004	0.022 ± 0.001	0.092 ± 0.005	0.031 ± 0.002	0.083 ± 0.004	0.022 ± 0.001	0.084 ± 0.005	0.035 ± 0.003
M2 (171 h)	0.109 ± 0.003	0.035 ± 0.001	0.089 ± 0.004	0.026 ± 0.004	0.078 ± 0.004	0.023 ± 0.001	0.108 ± 0.006	0.034 ± 0.002	0.087 ± 0.003	0.024 ± 0.001	0.093 ± 0.008	0.036 ± 0.004
M3 (200 h)	0.124 ± 0.006	0.042 ± 0.001	0.093 ± 0.006	0.031 ± 0.001	0.086 ± 0.002	0.032 ± 0.003	0.119 ± 0.007	0.039 ± 0.002	0.088 ± 0.005	0.029 ± 0.002	0.111 ± 0.007	0.040 ± 0.003
P1 (226 h)	0.129 ± 0.009	0.046 ± 0.003	0.106 ± 0.004	0.039 ± 0.002	0.095 ± 0.002	0.040 ± 0.002	0.118 ± 0.003	0.044 ± 0.003	0.093 ± 0.004	0.033 ± 0.001	0.119 ± 0.008	0.046 ± 0.003
P5 (346 h)	0.146 ± 0.007	0.059 ± 0.004	0.123* ± 0.008	0.046* ± 0.004	0.108* ± 0.005	0.048* ± 0.004	0.134 ± 0.006	0.059 ± 0.003	0.105* ± 0.006	0.04* ± 0.002	0.127 ± 0.006	0.053 ± 0.004

* *P* < 0.05

### Microbial richness and diversity

**Fig. 2:**
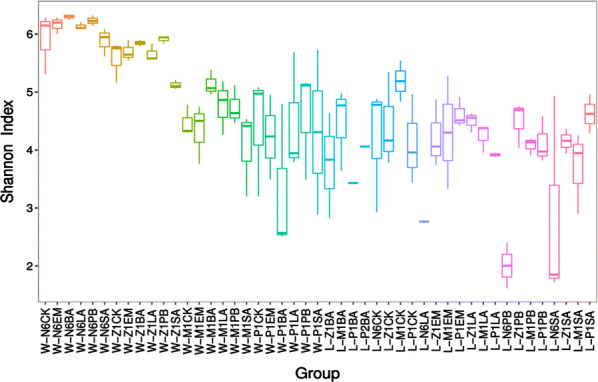
Shannon index of the rearing the rearing water and the larvae from N6 to P1 developmental phase with five probiotics treatment. The horizontal coordinate was marked with the group name and the vertical coordinate was the index value of Shannon. Each box plot showed the minimum index value, first quartile, median, third quartile and maximum value of the samples in the group, respectively

**Fig. 3:**
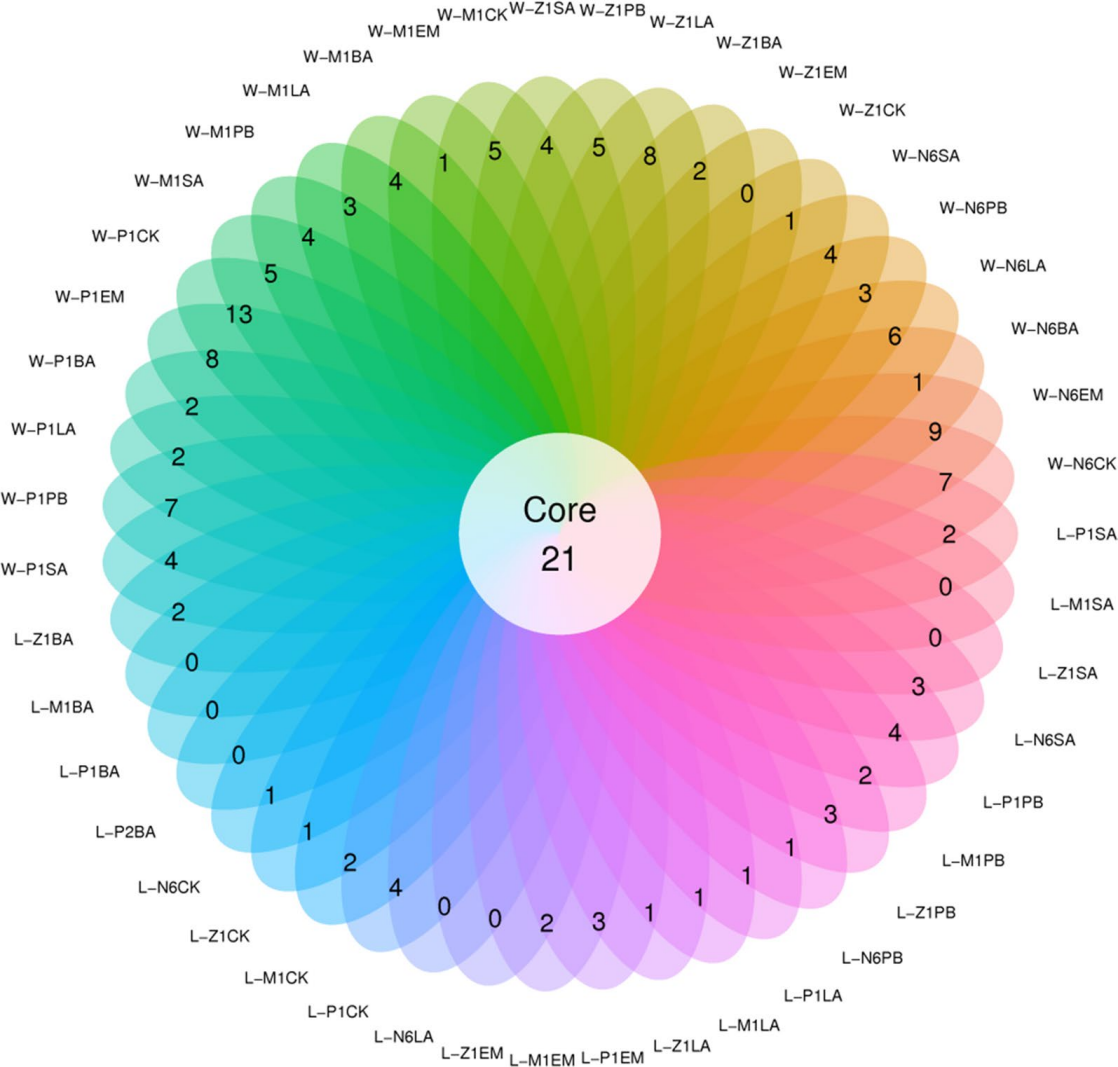
OTUs Venn diagram (or Petal diagram) in the larvae and the rearing water from N6 to P1 developmental phase with five probiotics treatment. Different color rings in the Venn diagram means different samples, and the number in every ring represented the number of unique OTUs (or species) in each sample, the number in the middle white circle represented the same OTUs shared among all samples

The Illumina MiSeq sequencing platform yielded in total 8112239 reads over all 131 samples (the Illumina MiSeq sequencing raw data were in the NCBI Sequence Read Archive database and the submitted no. was PRJNA614602, detailed in https://www.ncbi.nlm.nih.gov/sra/). Boxplot of differences between Shannon index groups were shown in Fig. 2. The result of baterial diversity analysis in rearing water was reflected by Shannon index as follow: during the N6 period, the Shannon index of all the experimental groups were higher than that of the control groups except for LA-treated groups. By Z1 stage, the Shannon index of the PB-treated and BA-treated groups were higher than that of the controls in the water samples (Fig. 2). When reach M1 stage, Shannon index of all the experimental groups were higher than the control groups. Interestingly, the variety of bacterial diversity was very great while got to P1 stage. All experimental groups were much lower than the control group except for SA-treated group. And the change of PB-treated group was the most significant, which suggested the unstable appearance. Bacterial diversity in the larvae at different stages had also been analyzed in the present study. It suggested that even with same probiotic treatment, there were differences in the bacterial flora in the larvae different development stages. Obviously, compared with the Shannon index in the rearing water, the bacterial diversity in shrimp larvae were much lower in N6 period. Results (Fig. 3) showed that, compared with the microflora in the shrimp larvae, microflora in rearing water environment was much more variable. It was showed that the sequence decreasingly of the unique OTUs (or species) were sample W-P1CK (13 uniques),W-N6EM (9 uniques),W-Z1LA (8 uniques), W-P1EM (8 uniques), W-P1PB (7 uniques), W- N6LA (6 uniques), and W-Z1PB, W-M1CK, W-M1SA (were all 5 uniques).

### Succession of bacterial community and the keystone species

**Fig. 4:**
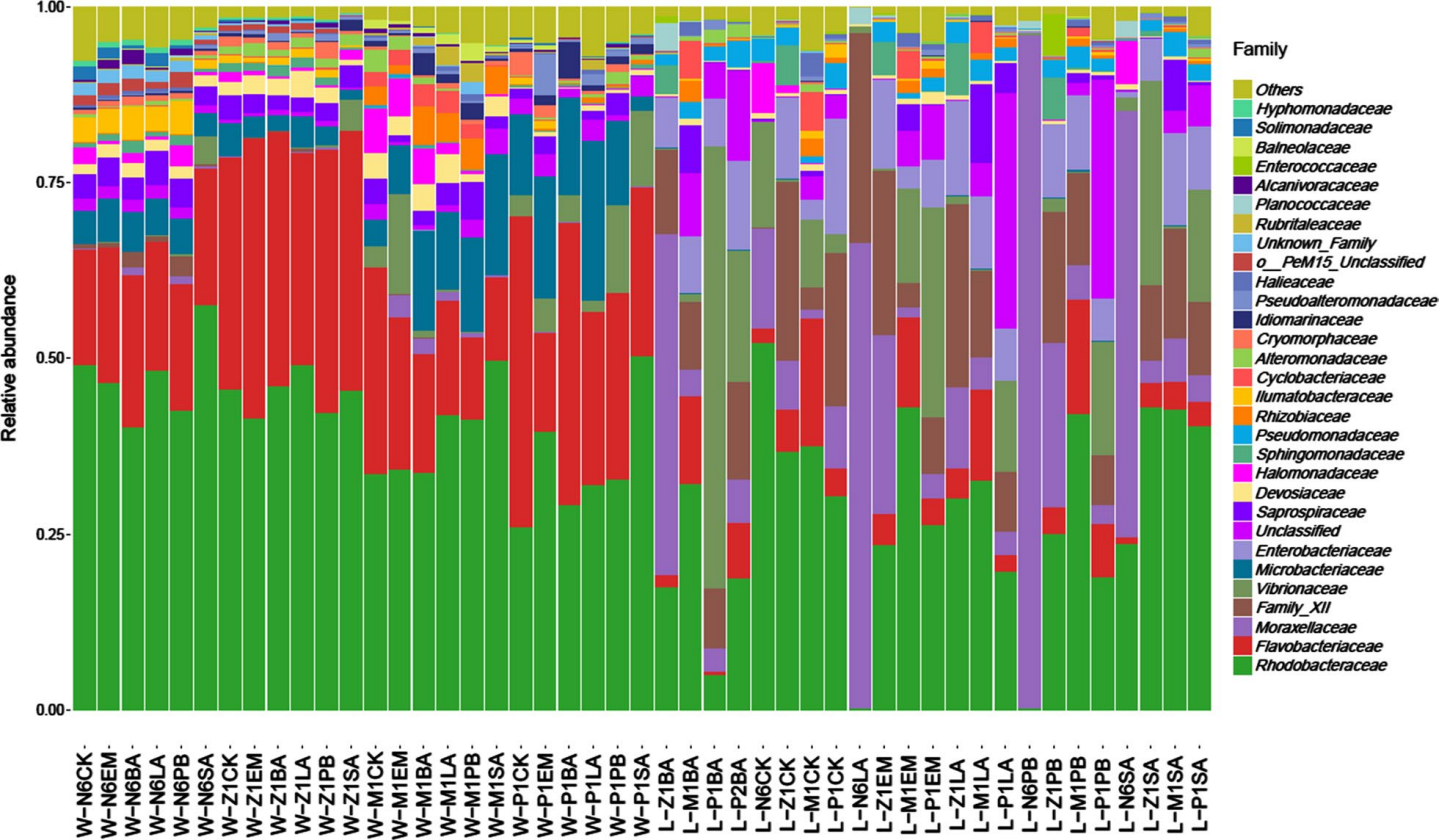
Histogram of bacteria families distribution for each sample in the larvae and the rearing water from N6 to P1 developmental phase with five probiotics treatment. The abscissa were the sample names, the ordinate was relative abundance of different species, the legend was the species classification names of different classification levels, and other represents the relative abundance and relative abundance of all other phylum levels except the first 30 relative abundance

The composition of the bacterial community in the larvae and the rearing water environment from N6 stage to P1 stage were studied integrally. The main abundant bacteria were analyzed at the family level (Fig. 4). It was showed that *Rhodobacteraceae* (the peack was 61.88% in W-N6SA) and *Flavobacteriaceae* (the peak was 67.46% in W-P1CK) were the dominant bacteria in almost all the samples, including larvae-derived and water-derived samples from N6 to P1 stage with five probiotics treatment, expected for L-N6LA, L-N6PB, L-Z1BA, L-P1BA, which was absolutely dominated by *Moraxellaceae* or *Vibrionaceae*. In comparison, family diversity of the water-derived bacteria were much richer than the the larvae-derived bacteria. In the rearing water during the development stage, *Microbacteriaceae*, *Vibrionaceae*, *Saprospiraceae*, *Devosiaceae*, *Halomonadaceae*, *Ilumatobacteraceae* and *Rhizobiaceae* were and these bacteria accounted for more than 80% of the bacteria. Notably, *Microbacteriaceae* was the unique in the rearing water, but absented in the larvae. Moreover, it was showed that the main difference of the bacterial community in water environment after five different probiotics treatment was changes of the ratio of dominant species, but the bacterial species were unchanged, approximately (Fig. 4). In comparison, the bacterial species varied greatly and there were fewer species in the larvae. In addition to the dominant bacteria including *Rhodobacteraceae*, *Flavobacteriaceae*, *Moraxellaceae* and *Vibrionaceae* in the larvae, other bacterias were also frequently-occurring, such as *Enterobacteriaceae*, *Saprospiraceae*, *Bacillales* Family_XII, *Halomonadaceae*, *Pseudomonadaceae*, *Rhizobiaceae* etc. Figure 4 suggested that there were no regular differences among the bacterial community of the larvae after five different probiotics treatment.

**Fig. 5:**
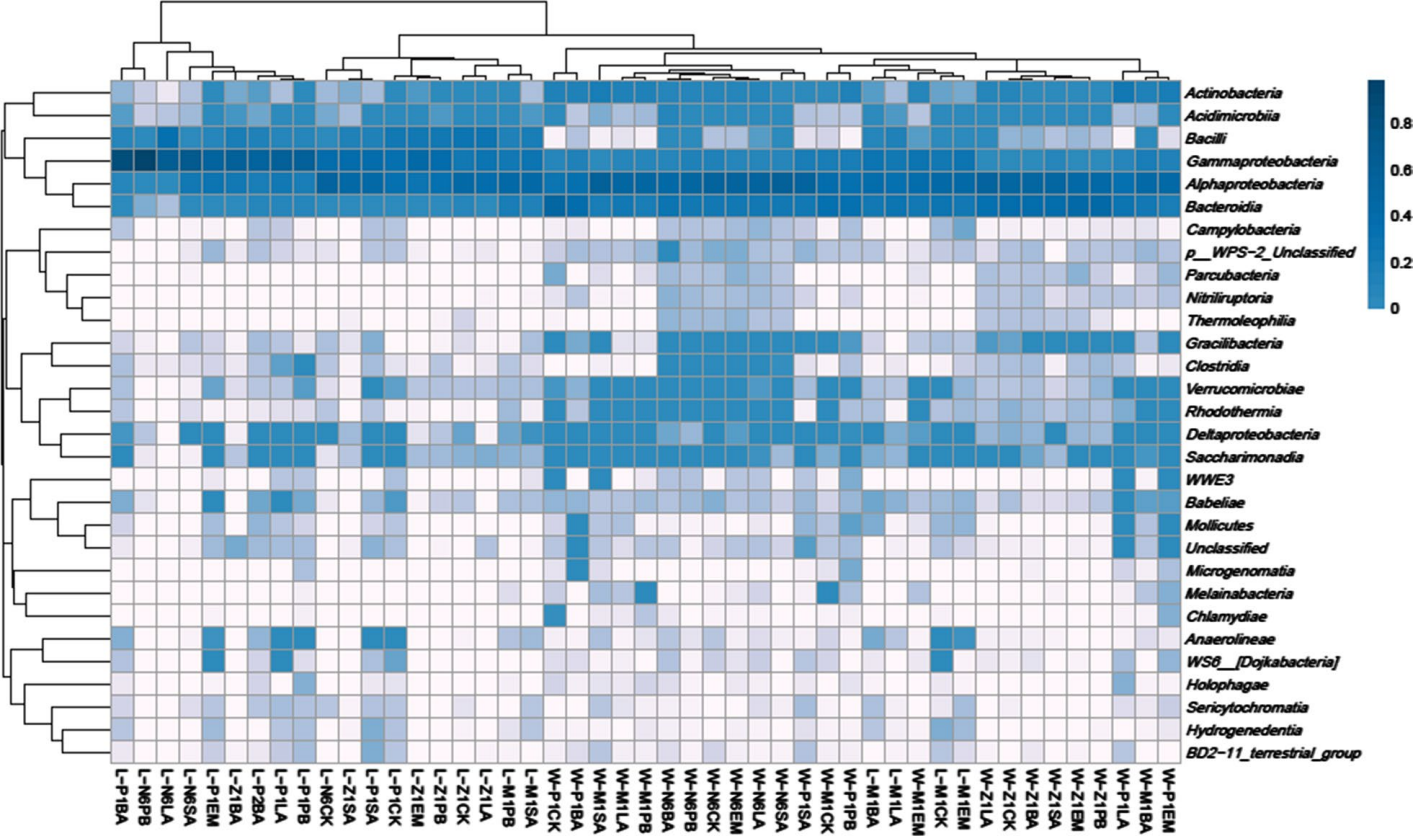
Heat-map display for bacteria families cluster in the larvae and the rearing water from N6 to P1 stage with five probiotics treatment. The row names were bacteria families, the column name was sample information, the left side of the figure were bacteria families cluster tree, the top was samples cluster tree, and the value corresponding to the color of each square in the middle heat map was the value of relative abundance of bacteria families abundance in each row after normalization

20 constant microflora were shared among all the samples from different probiotics-treated environment and shrimp larvae, from different developmental stages. The heat-map display for OTUs abundance cluster (Fig. 5) had shown the degree of enrichment of each bacterium. Mostly, bacterial flora in the larvae or from the rearing water environment were separate, regardless of the different probiotics treatment. But at the M1 stage, bacterial flora in the larvae and in the environment were similar. M1 stage is the key period of feeding transformation, which is from algophagous feeding becomes carnivorous feeding. Thus, the bacterial flora became unstable. Results suggested that the flora of larvae in this period was more susceptible to the flora in the water environment.

**Fig. 6:**
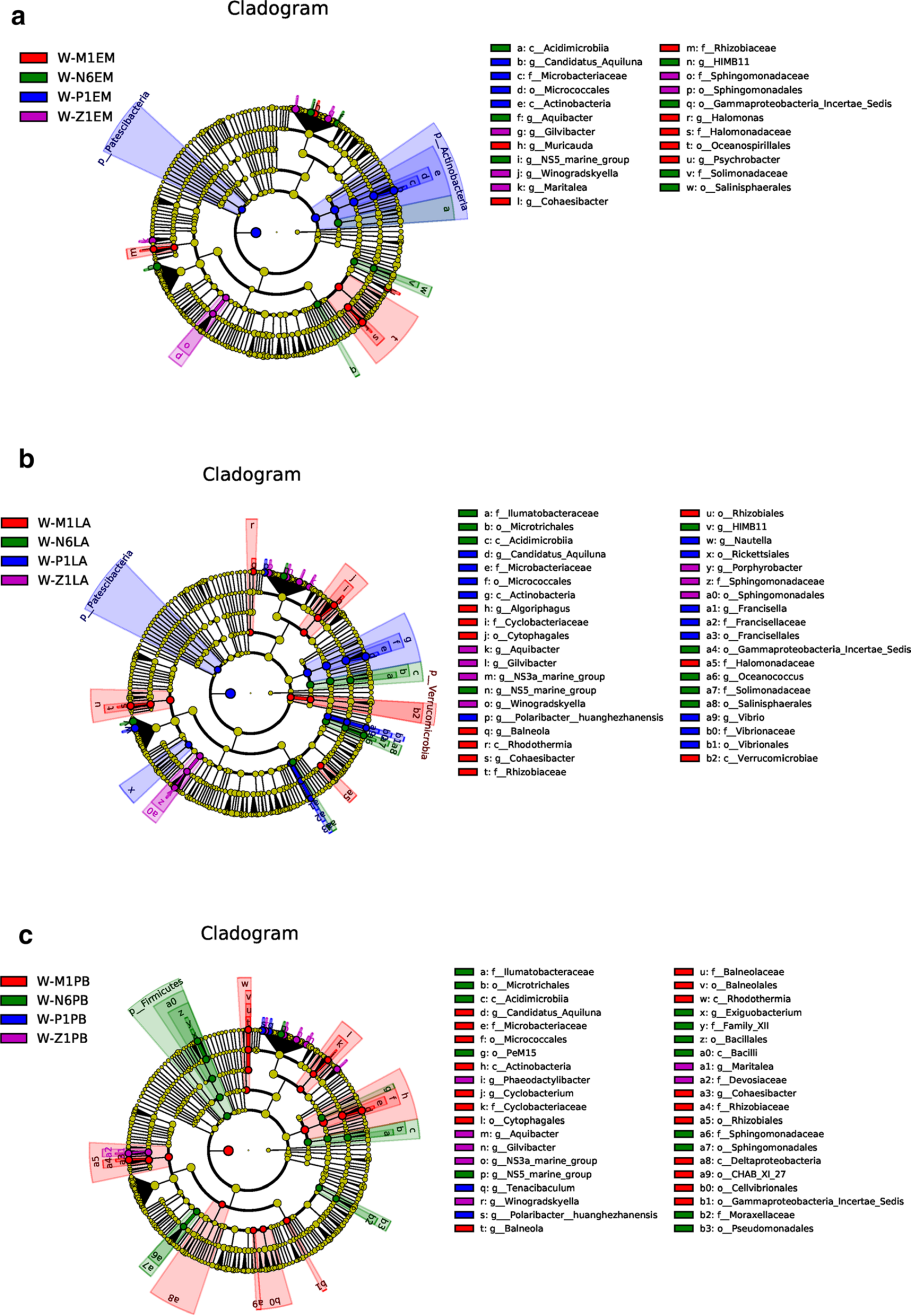
LefSe (LDA effect size) analysis for the specific dominant bacteria at different developmental stages with EM, LA or PB treatment. The circles radiating from inside to outside represent the classification levels from phylum to genus. Each small circle at the same classification level represents a classification at that level, and the diameter of the circle represents the relative abundance. The populations with no significant differences were uniformly stained yellow, and the biomarkers with significant differences were stained with the groups, such as the blue nodes represent the microbial groups that play an important role in the blue group, etc.

The data above, including the survival rate, metamorphosis rate and quantity of vibrios, showed that EM, LA and PB had better effect. Thus, the specific dominant bacteria at different developmental stages was concerned. It suggested that the dominant bacterial flora of the EM and LA treatment were similar. At the P1 stage, which was the critical period of morphogenesis for larvae, the main bacteria were *Microbacteriaceae* and *Rhizobiaceae* in the rearing water of EM-treated group, *Microbacteriaceae* and *Polaribacter* at LA-treated group, and *Tenacibaculum* and *Polaribacter* at PB-treated group (Fig. 6a–c).

## Discussion

Probiotics have been defined as “Live microorganisms (bacteria or yeasts), which when ingested or locally applied in sufficient numbers confer one or more specified demonstrated health benefits for the host” (Anil and Harjinder 2007). In fact, probiotics have a long history of use for human (Li et al. 2014). Probiotics are getting more and more attention in promoting animal growth and prevention of diseases in aquaculture (Zhao et al. 2018), and adding probiotics to the feed or the breeding environment is always the main approach. Good application practice might masked adding probiotics is beneficial to improving water quality (Pacheco-Vega et al. 2018). The intestinal tract is a complex ecosystem, which is a haven for countless diverse bacterial populations that have been shown to have effects on the host’s immunity or nutrient processes (Rungrassamee et al. 2013).

In the present study, the information were collected through all experiments and data analysis. Firstly, the results of using probiotics in commercial shrimp farm was concluded. The experiment had shown the relationship between larval survival rate and present of probiotics during the important development stages. The result was positive correlation (*P *< 0.05), indicating that the survival rate of probiotics treatment groups from Z1 stage to P5 stage were higher than that of control group. For example, the survival rate of LA-treated group was 10.33% higher than that of the control group. This fact is also proved by the experiment through SPSS statistic program. The data analyzation suggested that LA bacteria could improve the larvae’s survival ability (*P *< 0.05), that was related to the seedling emergence rates. Especially, in the larval stages M2, M3, P1, P5, the survival rate values had increased 5–10%, comparing with control groups. It was probably because LA bacteria could promote the metabolism for the excess nutrient in the rearing water, and furtherly reduce the phenomenon of sticky legs, which is leading malnutrition of shrimp larval by limiting their feeding capacity and vitality. LA bacteria have gained much attention as probiotics in aquaculture (Ringø 2020). Secondly, the metamorphosis rate had been quantified and the result showed in a positive way. The data showed that the metamorphosis rate has increased by 10% compares with control group. This indicated probiotics could promote the growth and development of shrimp larvae and prevent the incomplete molting in their growing process, furtherly reduce the case of hypo-genesis and malformation. The positive effect of using probiotics on molting of shrimp larval has being confirmed through this experiment. The results of the EM treatment group had shown the most remarkable difference compared with the control group (*P *< 0.05). The metamorphosis rate of EM-treated group in every stages had increased 4.5% in average, and this value had raised from 76.0 to 86.0 in P1 stage. However, how the diverse probiotics act on larval molting process is not yet explained in the present study. Furthermore, the vibrio quantity was tested too. *Vibrio* spp. had long been a major pathogen that causing diseases of shellfish, especially shrimps. The reason why vibriosis can be so threatening is because they can hurt shrimps’ exoskeletons, which are important and primary barriers for shrimps to defense multiple etiological agents (Beshiru and Igbinosa 2018; Navaneeth et al. 2020). Generally, most pathogenic vibrios would cause diseases under a certain conditions, and in aquaculture, fighting infectious diseases is necessity (Anaya-Rosas et al. 2019). Overall, in this experiment, it suggested that the quantity of vibrios were rapidly reduced after adding probiotics in 8 h. Within 23 h, the values of all treated groups were beck up, and reached the similar value as the control group’s value in N6 stage. However, for the next stages, all probiotics-treated groups had shown a significant decreasing trend in the quantity of vibrios, excepted for the SA-treated group, which’s values were staying closely with the control group. In detail, the EM-treated group presented the lowest vibrios quantity comparing with control group in early life period from rearing water stage to M1(143 h) stage, but then the value tends to be average. In opposite, the vibrios quantity of LA-treated group was more stable, and the gap between the LA-treated group and the control group had increasing generally. Until P5 stage, EM and LA-treated group had reached a similar level at 3.05 approximately. The phenomenon of EM- and LA-treated groups had explained that, different probiotics could inhibit the proliferation of vibrios in different life periods. Previous resport had suggested that *Bacillus* probiotics were screened for their ability to control pathogenic *Vibrio* spp. (Kewcharoen and Srisapoome 2019). And in the present study, inhibitory effect to the quantity of vibrio of BA was intermediate, that the inhibitory effect was less than PB. Otherwise, results also indicated that concentration of NH_4_-N and NO_2_-N in the rearing water with probiotics had decreased, especially in the groups with EM, BA and PB were significantly lower than that in control group at the P5 stage (*P* < 0.05). It furtherly proved that the diversity of probiotics in aquatic environment is essential for shrimps, particularly for larvae. To sum up, when there is a situation that increase in biomass of water, adding probiotics can reduce the concentration of organic materials and ammonia. This process could also reduce the pathogens are which causing exoskeleton diseases. “This procedure was accomplished by a series of enzymatic process carried out in succession by the various strains present in the probiotic blend. The addition of this blend to the cultured systems reduced the concentration of *Vibrio* strains and thus controlled diseases caused by *Vibrio* strains” (Farzanfar 2006). Among these probiotics, LA, EM and PB bacteria had shown the best effect, including improving survival rate of the larvae, promoting the larval metamorphosis, reducing the quantity of vibrios significantly and inhibiting NH_4_-N and NO_2_-N levels. And the unique dominant bacterial flora in the three probiotics treated groups were mainly *Microbacteriaceae*, *Rhizobiaceae*, *Polaribacter* and *Tenacibaculum*.

This reflects to the next step of the research, the examination of diversity of bacteria in tested samples. For getting the information, Shannon index value has been utilized. This index shows the median diversity of bacteria of overall 131 samples. The larger Shannon index means the more even or better proportion of the bacteria species in each group. By analyzing the index value, it has been found that the diversity of bacteria is unstable throughout larval stages. In addition, the diversity values of sample groups were within a wide range during P1 stage which means the bacterial diversity was unstable and unpredictable in this stage. Moreover, the heat-map display for OTUs abundance cluster has shown the degree of enrichment of each bacterium. In general, *Actinobacteria*, *Acidimicrobiia*, *Bacilli*, *Gammaproteobacteria*, *Alphaproteobacteria*, and *Bacteroidia* were often occur in all larval stages, especially the last three kind. Also, the heat-map of species distribution had shown the relative abundance of bacterial families. The most abundant family was *Rhodobacteraceae*, the second was *Flavobacteriaceae* and the third was *Moraxellaceae*. Firstly, *Rhodobacteraceae* was always the most abundance family in all larval stages except P1CK, P1BA, Z1BA, N6LA, Z1EM, N6PB, Z1PB, P1PB, N6SA. Secondly, *Flavobacteriaceae* was abundant during N6, Z1, M1, and P1 stages, which suggested *Flavobacteriaceae* was common bacterial flora in seawater. Thirdly, *Moraxellaceae* was the most abundant bacteria in Z1BA, N6LA, N6PB, and N6SA stages. This result indicated that *Moraxellaceae* was the main microflora during the nauplius stage, and also implied *Moraxellaceae* might be the typical bacteria in the maternal broodstock. Besides, *Vibrionaceae* was also a common flora in the larvae, no matter which probiotics had been used. And once *Vibrionaceae* appeared, the proportion was also high, such as SA-treated at Z1 stage, BA-treated at P1 stage and EM-treated at P1 stage for larvae, etc. So, attention needs to be paid in these stages. Noteworthily, all the probiotics added externally did not become the dominant flora, even did not last long in the rearing water.

Thus, depends on the existing researches, in an appropriate species richness and species diverse aquatic environment, the water system is more likely to be healthy since the the high bacterial diversity could be related with the numerous biological processes influencing the consumption of organic matter and the transformation of nitrogen compounds (Huerta-Rábago, et al. 2019).Obviously, the present study suggested exogenous probiotics had a little impact on the community of indigenous bacteria, which could still keep multiple bacterial flora co-existing, this was beneficial to maintain the stability of the whole aquatic environment and avoid the stress response of larvae. What regulates the balance of the bacterial community, even introducing foreign probiotics to the water environment? Is it a competition for nutrition? Or the dominant bacteria in the environment? Further analysis through metagenomic techniques should be required in future.

## Data Availability

All data generated or analysed during this study are included in this published article.

## References

[CR1] Adel M, El-Sayed AFM, Yeganeh S, Dadar M, Giri SS (2017). Effect of probiotic *Lactococcus lactis* subsp. *lactis* on growth performance, intestinal microbiota, digestive enzyme activities, and disease resistance of *Litopenaeus vannamei*. Probiotics Antimicrobiol.

[CR2] Aguilera-Rivera D, Prieto-Davó A, Escalante K, Chávez C, Cuzon G, Gaxiola G (2014). Probiotic effect of FLOC on *Vibrios* in the pacific white shrimp *Litopenaeus vannamei*. Aquaculture.

[CR3] Anaya-Rosas RE, Rivas-Vega ME, Miranda-Baeza A, Piña-Valdez P, Nieves-Soto M (2019). Effects of a co-culture of marine algae and shrimp (*Litopenaeus vannamei*) on the growth, survival and immune response of shrimp infected with *Vibrio parahaemolyticus* and white spot virus (WSSV). Fish Shellfish Immunol.

[CR4] Anil KA, Harjinder S (2007). Recent advances in microencapsulation of probiotics for industrial applications and targeted delivery. Trends Food Sci Technol.

[CR5] Arias-Moscoso JL, Espinoza-Barrón LG, Miranda-Baeza A, Rivas-Vega ME, Nieves-Soto M (2018). Effect of commercial probiotics addition in a biofloc shrimp farm during the nursery phase in zero water exchange. Aquacult Rep.

[CR6] Beshiru A, Igbinosa EO (2018). Characterization of extracellular virulence properties and biofilm-formation capacity of *Vibrio* species recovered from ready-to-eat (RTE) shrimps. Microb Pathogenesis.

[CR7] Chai PC, Song XL, Chen GF, Xu H, Huang J (2016). Dietary supplementation of probiotic *Bacillus* PC465 isolated from the gut of *Fenneropenaeus chinensis* improves the health status and resistance of *Litopenaeus vannamei* against white spot syndrome virus. Fish Shellfish Immunol.

[CR8] Chaiyapechara S, Rungrassamee W, Suriyachay I, Kuncharin Y, Klanchui A, Karoonuthaisiri N, Jiravanichpaisal P (2012). Bacterial community associated with the intestinal tract of *P. monodon* in commercial farms. Microb Ecol.

[CR9] Chumpol S, Kantachote D, Nitoda T, Kanzaki H (2018). Administration of purple nonsulfur bacteria as single cell protein by mixing with shrimp feed to enhance growth, immune response and survival in white shrimp (*Litopenaeus vannamei*) cultivation. Aquaculture.

[CR10] De la Banda IG, Lobo C, Chabrillon M, Leon-Rubio JM, Arijo S, Pazos G, Lucas LM, Morinigo MA (2012). Influence of dietary administration of a probiotic strain *Shewanella putrefaciens* on *Senegalese sole* (*Solea senegalensis*, Kaup 1858) growth, body composition and resistance to *Photobacterium damselae* subsp *piscicida*. Aquacult Res.

[CR11] Douillet PA, Langdon CJ (1994). Use of a probiotic for the culture of larvae of the Pacific oyster (*Crassostrea gigas* Thunberg). Aquaculture.

[CR12] Emerenciano MGC, Martínez-Córdova LR, Martínez-Porchas M, Miranda-Baeza A (2017). Biofloc technology (BFT): a tool for Water quality management in aquaculture. In: Tutu H (ed.) Water quality, Intech, pp 91–109

[CR13] Farzanfar A (2006). The use of probiotics in shrimp aquaculture. FEMS Immunol Med Microbiol.

[CR14] Food Agric Organ United Nations, FAO (2001). Evaluation of health and nutritional properties of powdered milk and live lactic acid bacteria. Expert consultation report.

[CR16] Food Agric Organ United Nations, FAO (2016). The State of World Fisheries and Aquaculture 2016. Contributing to food security and nutrition for all.

[CR17] Huang ZB, Li XY, Wang LP, Shao ZZ (2016). Changes in the intestinal bacterial community during the growth of white shrimp, *Litopenaeus vannamei*. Aquacult Res.

[CR18] Huerta-Rábago JA, Martinez-Porchas M, Miranda-Baeza A, Nieves-Soto ME, Martinez-Cordova LR (2019). Addition of commercial probiotic in a biofloc shrimp farm of *Litopenaeus Vannamei* during the Nursery phase: effect on bacterial diversity using massive sequencing 16S rRNA. Aquaculture.

[CR19] Kewcharoen W, Srisapoome P (2019). Probiotic effects of *Bacillus* spp. from Pacific white shrimp (*Litopenaeus vannamei*) on water quality and shrimp growth, immune responses, and resistance to *Vibrio parahaemolyticus* (AHPND strains). Fish Shellfish Immunol.

[CR20] Kim DH, Brunt J, Austin B (2007). Microbial diversity of intestinal contents and mucus in rainbow trout (*Oncorhynchus mykiss*). J Appl Microbiol.

[CR21] Krummenauer D, Poersch L, Romano LA, Lara GR, Encarnação P, Wasielesky W (2014). The effect of probiotics in a *Litopenaeus vannamei* biofloc culture system infected with *Vibrio parahaemolyticus*. J Appl Aquacult.

[CR22] Li GY, Song XL, Sun Y (2011). Effects of probiotics from the shrimp intestine on the non-specific immunity and antiviral capacity of *Litopenaeus vannamei*. J Fish Sci China.

[CR23] Li YB, Xu QQ, Yang CJ, Yang X, Lv L, Yin CH, Liu XL, Yan H (2014). Effects of probiotics on the growth performance and intestinal micro flora of broiler chickens. Pak J Pharm Sci.

[CR24] López-Elías JA, Moreno-Arias A, Miranda-Baeza A, Martínez-Córdova LR, Rivas-Vega ME, Márquez-Ríos E (2015). Proximate composition of bioflocs in culture systems containing hybrid red tilapia fed diets with varying levels of vegetable meal inclusion. N Am J Aquac.

[CR25] Martínez-Córdova LR, Martínez-Porchas M, Emerenciano MGC, Miranda-Baeza A, Gollas-Galván T (2017). From microbes to fish the next revolution in food production. Crit Rev Biotechnol.

[CR26] Moreno-Arias A, López-Elías JA, Martínez-Córdova LR, Ramírez-Suárez JC, Carvallo-Ruiz MG, García-Sánchez G, Lugo-Sánchez ME, Miranda-Baeza A (2018). Effect of fishmeal replacement with a vegetable protein mixture on the amino acid and fatty acid profiles of diets, biofloc and shrimp cultured in BFT system. Aquaculture.

[CR27] Navaneeth KA, Bhuvaneswari T, RajanS JJS, AlavandiKSV Vijayan KK, Otta SK (2020). Characterization of *Vibrio parahaemolyticus* isolates from shrimp farms of Southeast coast of India with special reference to Acute Hepatopancreatic Necrosis Disease (AHPND) status. Aquaculture.

[CR28] Nimrat S, Boonthai T, Vuthiphandchai V (2011). Effects of probiotic forms, compositions of and mode of probiotic administration on rearing of Pacific white shrimp (*Litopenaeus vannamei*) larvae and postlarvae. Anim Feed Sci Technol.

[CR29] Niu YF, Defoirdt T, Rekecki A, Schryver PD, Broeck WVD, Dong SL, Sorgeloos P, Boon N, Bossier P (2012). A method for the specific detection of resident bacteria in brine shrimp larvae. J Microbiol Methods.

[CR30] Nogam IK, Maeda M (1992). Bacteria as biocontrol agents for rearing larvae of the crab *Portunus trituberculatus*. Can J Fish Aquat Sci.

[CR31] Oetama VSP, Hennersdorf P, Abdul-Aziz MA, Mrotzek G, Haryanti H, Saluz HP (2016). Microbiome analysis and detection of pathogenic bacteria of *Penaeus monodon* from Jakarta Bay and Bali. Mar Pollut Bull.

[CR32] Pacheco-Vega JM, Cadena-Roa MA, Leyva-Flores JA, Zavala-Leal OI, PérezBravo E, Ruiz-Velazco JMJ (2018). Effect of isolated bacteria and microalgae on the biofloc characteristics in the Pacific white shrimp culture. Aquacult Rep.

[CR33] Qiu BS, Lin WT, Yang JG, Wu JL (2004). Application of probiotics in aquaculture. Fish Sci.

[CR34] Ringø E (2020). Probiotics in shellfish aquaculture. Aquacult Fish.

[CR35] Roeselers G, Mittge EK, Stephens WZ, Parichy DM, Cavanaugh CM, Guillemin K, Rawls JF (2011). Evidence for a core gut microbiota in the zebrafish. ISME J.

[CR36] Rungrassamee W, Klanchui A, Chaiyapechara S, Maibunkaew S, Tangphatsornruang S, Jiravanichpaisal P, Karoonuthaisiri N (2013). Bacterial population in intestines of the black tiger shrimp (*Penaeus monodon*) under different growth stages. PLoS ONE.

[CR37] Verschuere L, Rombaut G, Sorgeloos P, Verstraete W (2000). Probiotic bacteria as biological control agents in aquaculture. Microbiol Mol Biol Rev.

[CR38] Wang XE, Sun ZX, Liu XY, Ma JX, Wang BS, Song XJ, Zhu ZW, Shi JY, Wang FG (1994). Application of phototrophic bacteria in scallop artifical seed-breeding. J fisheries China.

[CR39] Wang RX, Wang JY, Sun YC, Yang BL, Wang AL (2015). Antibiotic resistance monitoring in *Vibrio* spp. isolated from rearing environment and intestines of abalone *Haliotis diversicolor*. Mar Pollut Bull.

[CR40] Wang RX, He J, Wang JY (2016). Heterotrophic bacterial abundance and diversity in the farming environment and guts of the oyster *Crassostrea hongkongensis*. J Shellfish Res.

[CR41] Wang RX, Yao T, Liu XJ, Wang JY (2018). Isolation and characterisation of *Vibrio harveyi* as etiological agent of foot pustule disease in the abalone *Haliotis discus hannai* Ino 1953. Indian J Fish.

[CR42] Yan BL, Wang XQ, Li SH, Wang DC, Dai Y, Shi DQ, Xu JT, Xu GC, Luo G (2005) Application of microbiological preparation to control of water quality in Fcatory Breeding of *Ericheir Sinensis*. Chin Agricult Sci Bull 21(3):329–333. http://zntb.chinajournal.net.cn

[CR43] Zhang M, Sun Y, Chen K, Yu N, Zhou Z, Chen L, Du Z, Li E (2014). Characterization of the intestinal microbiota in pacific white shrimp, *Litopenaeus vannamei*, fed diets with different lipid sources. Aquaculture.

[CR44] Zhao J, Ling YH, Zhang RZ, Ke C, Hong GL (2018). Effects of dietary supplementation of probiotics on growth, immune responses, and gut microbiome of the abalone *Haliotis diversicolor*. Aquaculture.

